# High-throughput sequencing of nematode communities from total soil DNA extractions

**DOI:** 10.1186/s12898-014-0034-4

**Published:** 2015-02-12

**Authors:** Rumakanta Sapkota, Mogens Nicolaisen

**Affiliations:** Department of Agroecology, Aarhus University, Faculty of Science and Technology, Forsøgsvej 1, DK-4200 Slagelse, Denmark

**Keywords:** Nematode, Community, Next-generation sequencing, SSU, Diversity, 18S, rDNA

## Abstract

**Background:**

Nematodes are extremely diverse and numbers of species are predicted to be more than a million. Studies on nematode diversity are difficult and laborious using classical methods and therefore high-throughput sequencing is an attractive alternative. Primers that have been used in previous sequence-based studies are not nematode specific but also amplify other groups of organisms such as fungi and plantae, and thus require a nematode enrichment step that may introduce biases.

**Results:**

In this study an amplification strategy which selectively amplifies a fragment of the SSU from nematodes without the need for enrichment was developed. Using this strategy on DNA templates from a set of 22 agricultural soils, we obtained 64.4% sequences of nematode origin in total, whereas the remaining sequences were almost entirely from other metazoans. The nematode sequences were derived from a broad taxonomic range and most sequences were from nematode taxa that have previously been found to be abundant in soil such as Tylenchida, Rhabditida, Dorylaimida, Triplonchida and Araeolaimida.

**Conclusions:**

Our amplification and sequencing strategy for assessing nematode diversity was able to collect a broad diversity without prior nematode enrichment and thus the method will be highly valuable in ecological studies of nematodes.

**Electronic supplementary material:**

The online version of this article (doi:10.1186/s12898-014-0034-4) contains supplementary material, which is available to authorized users.

## Background

More than one million species of nematodes are estimated to exist [1], but only a small fraction of this huge diversity has been described so far. Nematodes are among the most successful groups of animals: they are abundant, diverse and live in virtually all marine, freshwater and soil habitats. They occupy most trophic levels and play important roles in the soil ecosystem where they may cause large economic losses as parasites of animals and plants [2]. Total losses caused by plant-parasitic nematodes are estimated at $80 billion annually [3], and most of these, including cyst, lesion and root knot nematodes, belong to the order Tylenchida [4,5]. Furthermore, nematodes are suitable indicators of soil health as they are highly affected by nutrient status and the level of toxic compounds in the soil [6].

To overcome previous limitations in assessing nematode diversity, the efficiency of next-generation sequencing (NGS) technologies has been demonstrated. Porazinska *et al.* [7,8] sequenced a fragment of the ribosomal small subunit (SSU) and found that both qualitative and quantitative sequence data were consistent and reproducible using a nematode mock community. Studies of soil nematode communities by Morise *et al*. [9], Darby *et al*. [10] and Porazinska *et al*. [11], however, relied on enrichment of nematodes by sucrose flotation or by the Baermann funnel method to avoid amplification of DNA from other taxa that are abundant in soil such as fungi or plantae. This enrichment step is laborious and may be biased towards particular genera or developmental stages of nematodes.

We are using NGS to study soil communities and their effect on plant health and we are interested in different taxa such as fungi, oomycetes, bacteria and nematodes. In order to reduce biases and work load, primers that could selectively amplify different groups of organisms directly from one total soil DNA extraction would be desirable. Therefore, the aim of this study was to optimize a sequencing strategy that would allow us to study nematode diversity without the need for a nematode enrichment step. We tested the developed amplification strategy using total DNA from a number of agricultural soils as template.

## Results

**Figure 1:**
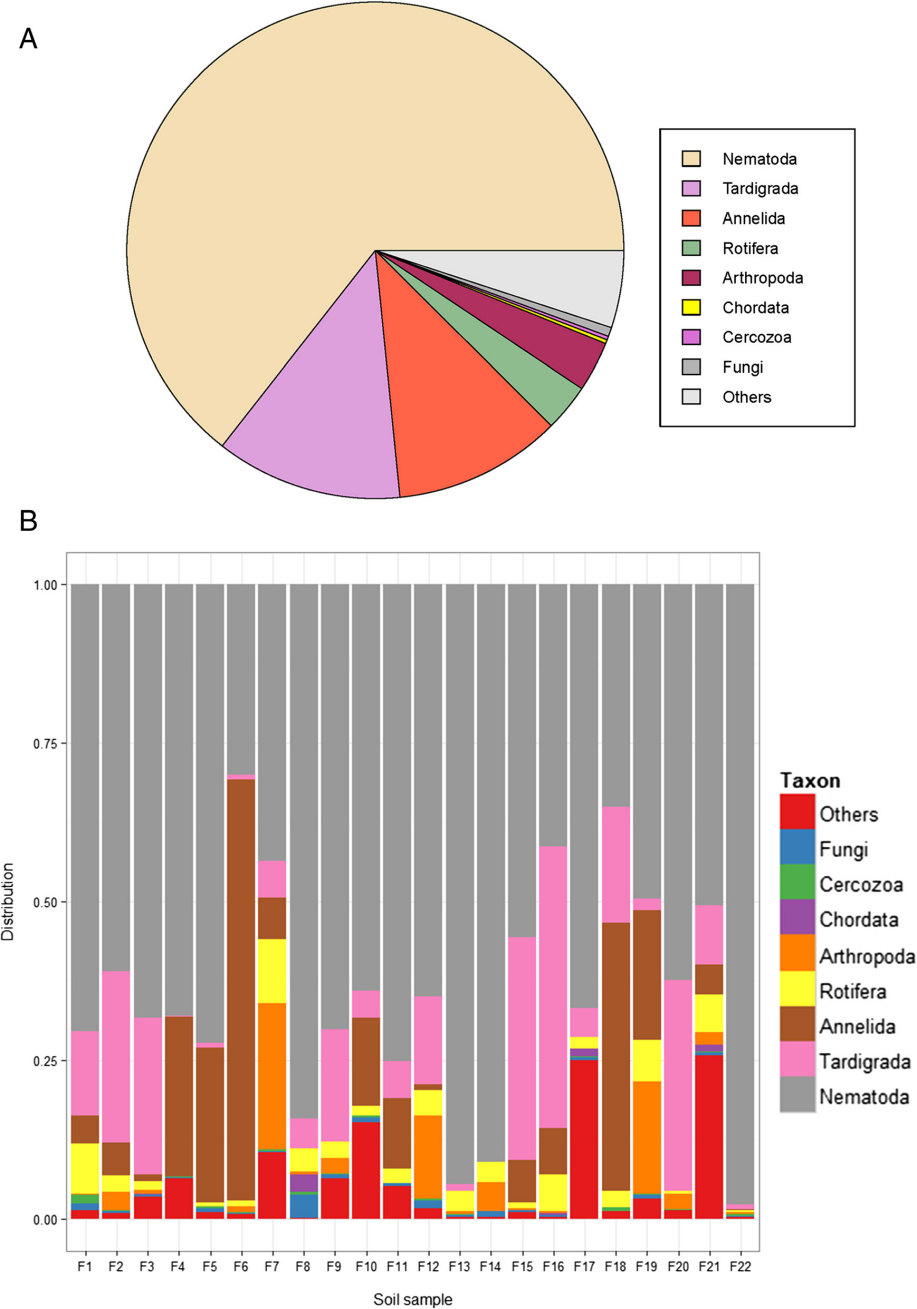
**The relative distribution of sequences in the total dataset at phylum rank (fungi at kingdom rank) for all soils (A) and for each of the 22 analyzed soils (B).**

An *in silico* analysis indicated that PCR amplification of DNA extracted from soil using the primers NF1 and 18Sr2b would potentially amplify not only nematodes but also other metazoans, plantae and fungi, although the primers have been used previously in several metagenetic studies of nematode communities [7,8,11]. In an initial experiment, we confirmed this by using these primers to amplify total DNA extracted from soil, without any steps to enrich for nematodes. Sequencing this amplicon resulted in only 3% sequences of nematode origin whereas the remaining sequences belonged to fungi, plants, rhizaria and metazoans (other than nematodes) (Additional file 1: Table S1 and unpublished observations). To obtain a higher proportion of nematode sequences without the need for nematode enrichment, we designed a forward primer aimed at being specific for nematode DNA amplification and used this in a semi-nested amplification strategy with NF1 and 18Sr2b. This strategy was tested using total DNA extracted from 22 agricultural soils from different areas of Denmark to evaluate the consistency of the amplification protocol. In total, 136,441 quality-filtered sequences were obtained and these could be clustered into 541 OTUs at 99% similarity. Of the total number of sequences, 64.4% were classified as belonging to Nematoda. The remaining sequences were dominated by Tardigrada (12.1%), Annelida (10.9%), Arthropoda (mainly Collembola and Arachnida) (3.3%) and Rotifera (3.1%) and only very few sequences that could be classified as belonging to plantae (0.1%) or fungi (0.6%) (Figure 1a). The remaining 4.1% were unclassified (Additional file 2: Table S2). In individual soils, between 30 and 97% of sequences were of nematode origin (Figure 1b). In the few soils with a relatively high amount of sequences belonging to other taxa such as Tardigrada or Annelida, these were usually dominated by one group (Additional file 2: Table S2).

**Figure 2:**
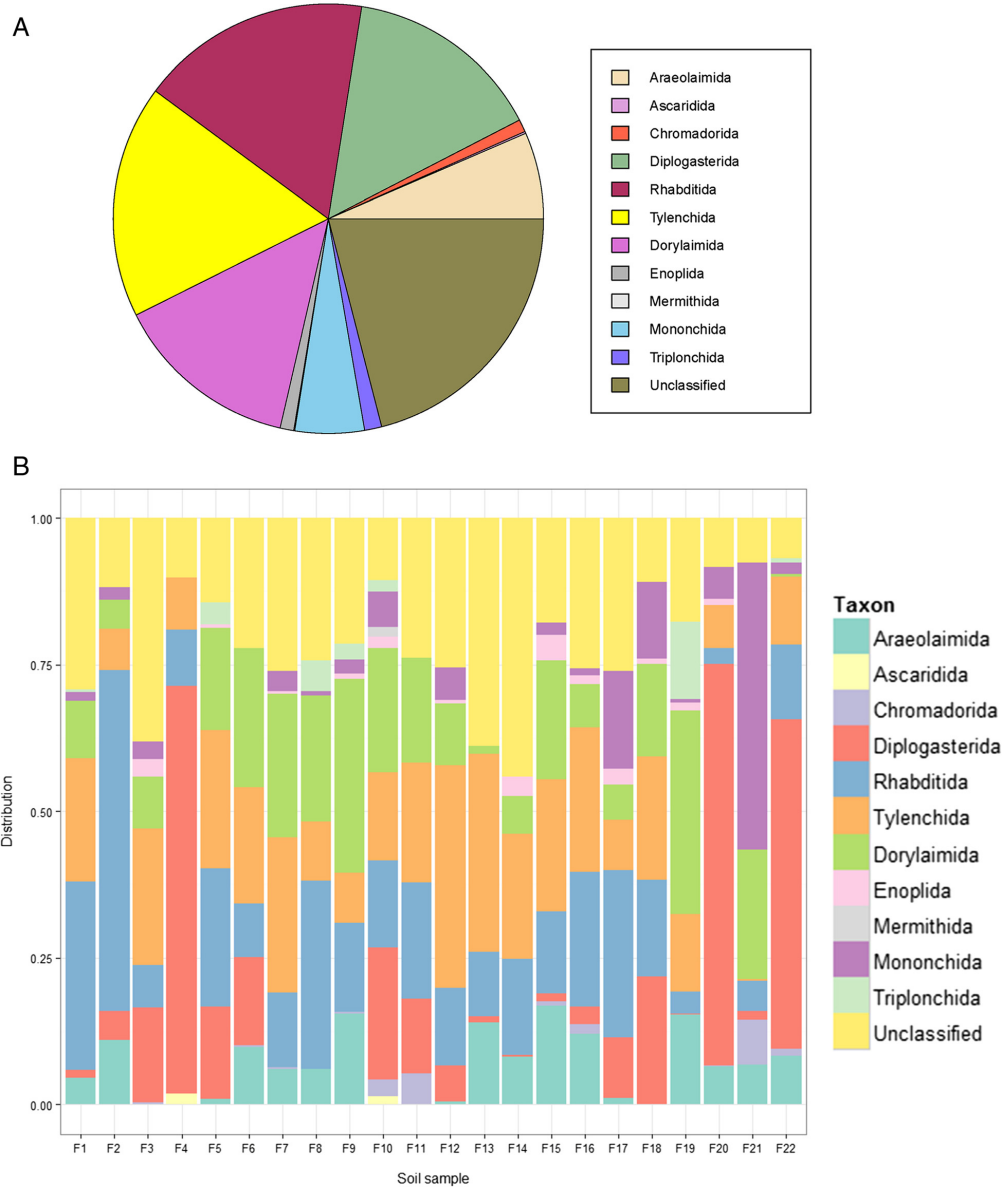
**The relative distribution of sequences in the nematode dataset within different nematode orders for all soils (A) and for each of the 22 analyzed soils (B).**

A taxonomic classification of the nematodes using the Silva 108 release as the reference set in QIIME [12] showed that a broad diversity of nematode taxa had been captured (Figure 2a, Additional file 3: Table S3). The taxa represent most of the orders defined in Blaxter *et al*. [13] except that we did not find OTUs belonging to orders such as Strongylida, Spirurida or Oxyurida, members which are parasites in vertebrates. Almost all orders of nematodes could be found in all soils, however, there was a noticeable variation in the relative composition of nematode groups among the individual soils from 22 agricultural fields (Figure 2b, Additional file 3: Table S3).

**Figure 3:**
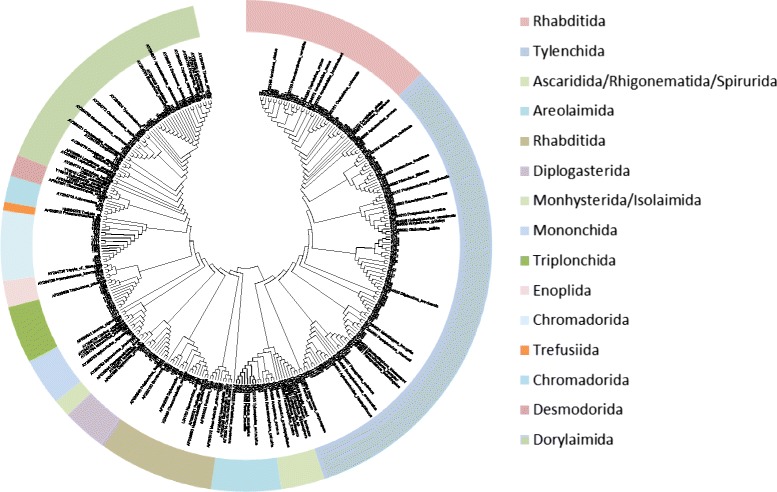
**Neighbor-joining tree of SSU rDNA barcode sequences illustrating the phylogenetic relationship for OTUs in the soil dataset (denoted with the OTU number) together with reference sequences (denoted with species name and GenBank accession number) covering most taxonomic groups within Nematoda.** For simplicity, bootstrap values are not shown, and the tree is shown with topology only. Nematode orders are indicated with different colors.

To confirm the broad distribution of sequences among taxa, we picked a representative sequence from each OTU (Additional file 4: Table S4) and used these sequences together with a reference set of GenBank sequences assembled by Morise *et al.* [9] to construct a phylogenetic tree. As can be seen from Figure 3, sequences generated in this study were distributed among the main taxonomic groups of nematodes. Most OTUs clustered in Tylenchida, Rhabditida, Dorylaimida, Triplonchida and Araeolaimida, but some of the OTUs were clustering within Mermithida, Mononchida, Diplogasterida, Enoplida, Chromadora, Desmodorida, Monhysterida, Rhigonematida and Ascaridida.

## Discussion

Traditionally, nematodes are enriched by e.g. sucrose flotation (e.g. [10]) or by using the Baermann funnel method [14] before microscopy and morphological identification, and even before most molecular analyses. However, this may not be practical when several groups of organisms such as nematodes, fungi and bacteria are of interest from one sample. In such cases one DNA extraction for all groups of organisms is desirable and allows direct comparisons between taxa. Furthermore, an enrichment step may introduce biases as particular nematode taxa or developmental stages are not necessarily enriched at the same efficiency, depending on the method used [15]. In a laboratory dealing with numerous samples, enrichment may easily become a bottleneck in the workflow and may require specialized equipment and expertise.

Currently used primers for studying nematode diversity also amplify fungi, plants and other metazoa from soil and therefore require an enrichment step to increase the proportion of nematode sequences. To overcome these limitations, we have developed an amplification strategy, including a newly developed primer, that efficiently amplifies nematode DNA (and other metazoan DNA) while excluding the amplification of fungal and plant DNA. When used on DNA extracted directly from 22 agricultural soils this amplification strategy resulted in 64.4% nematode sequences in total and very few plant or fungal sequences. The remaining 30% sequences were of other metazoan origin. In the individual soils, the proportion of nematode sequences varied from 30 to 97%. The relatively low proportion of nematode sequences in a few samples could generally be attributed to the dominance of one single group belonging to Annelida or Tardigrada (Additional file 2: Table S2).

According to the classification in QIIME using the Silva 108 reference set, the majority of sequences of nematode origin recovered in this study belonged to Rhabditida, Tylenchida, Diplogasterida, Dorylaimida and Araeolaimida, which is in general accordance with other studies of nematode diversity in agricultural soils using morphology (e.g. [16-19]) or sequencing [10] for identification (Additional file 1: Table S1), although a larger diversity is generally recovered in sequencing studies compared to classical morphology-based methods. There was a significant variation of nematode community structures between soils, probably reflecting different soil types, different crop plants and different agricultural practices. However, it was outside the scope of this study to compare nematode communities in the soils in detail.

To further investigate the diversity of nematode OTUs, a detailed survey of the taxonomic coverage of the assembled sequences was done by constructing a phylogenetic tree including a set of nematode reference sequences [9]. This tree confirmed that a broad taxonomic range of nematodes had been collected as sequences were found in all major branches of the tree. The highest numbers of OTUs were found to group with Tylenchida, Rhabditida, Dorylaimida, Triplonchida and Araeolaimida reference sequences. These are taxa that are known to include many plant parasites (Tylenchida, Dorylaimida, Triplonchida) or bacterivores (many Rhabditida) [13] and thus they are expected to be found in soil (Figure 3).

In our approach, we obtained a relatively large portion of metazoan sequences not belonging to Nematoda (Tardigrada (12.1%), Annelida (10.9%), Arthropoda (3.3%) and Rotifera (3.1%)); these sequences were disregarded as nematodes were the focus of this study. It remains to be tested whether the developed strategy can be used for studying this diversity too.

The development of new strategies for metagenetics often include sequencing of mock communities, however, we did not have access to a large collection of nematode specimens and thus we could not test our strategy on an assembled community. However, we found that our sequencing strategy recovered proportions of nematode taxa that were comparable to quantities obtained in an initial experiment using the previously published NF1/18Sr2b primer set (Additional file 1: Table S1). The distribution of nematode taxa in our study was also comparable to what has been observed in other studies using morphology for identification of nematodes from agricultural soils, indicating quantitative recovery. However, it has previously been shown that PCR based sequencing studies do not recover all species in a mock community quantitatively [8]. Within-sample comparisons of different nematode taxa may therefore be critical, whereas between-sample comparisons are probably still valid, as concluded by Amend *et al*. [20] using a fungal mock community and Porazinska *et al.* [8] using nematode mock communities.

## Conclusions

We have developed an amplification strategy, including a newly developed primer, for high-throughput sequencing. The strategy is efficient for studying nematode diversity in soil samples and most likely also from other habitats. The strategy does not require any nematode enrichment steps before PCR amplification, steps that might introduce biases in nematode sequence recovery. We show that by using this strategy, sequences from a broad range of nematode taxa, including economically relevant plant parasites, are recovered.

## Methods

### Primer design

We used a set of more than one thousand representative nematode SSU sequences compiled and analyzed in van Megen *et al.* [21], together with representative fungal, metazoan and plant SSUs to construct alignments using MEGA 5.22 [22]. Using these alignments we designed NemF (Table 1) that, in combination with 18Sr2b, was predicted to amplify only DNA from nematodes (and other metazoans), excluding fungal or plant DNA. The specificity is mostly based on the primer mismatch at the 3′ end to fungal and plant sequences (Table 2). Further, to obtain an amplicon with a size suitable for NGS, we used a semi-nested approach including the tagged primers NF1 and 18Sr2b in the final steps of the amplification; the specificity of these primers has been evaluated previously [15].

**Table 1:** **Primers used in this study**

**Primer**	**5′ – 3′ sequence**	**Reference**
NemF	GGGGAAGTATGGTTGCAAA	This study
NF1	GGTGGTGCATGGCCGTTCTTAGTT	[7]
18Sr2b	TACAAAGGGCAGGGACGTAAT	[7]

**Table 2:** **Specificity of the NemF primer**

**NemF**	**G**	**G**	**G**	**G**	**A**	**A**	**G**	**T**	**A**	**T**	**G**	**G**	**T**	**T**	**G**	**C**	**A**	**A**	**A**
Nematode	100	100	100	100	81	100	100	100	100	100	100	100	100	100	100	100	100	100	100
Fungi	.	.	.	.	**G**	.	.	.	.	.	.	.	.	**C**	.	.	.	.	**G**
Plantae	.	.	.	.	**G**	.	.	.	.	.	.	.	.	**C**	.	.	.	.	**G**
Metazoan	.	.	.	.	.	.	.	.	.	.	.	.	.	.	.	.	.	.	.

The first row below the NemF sequence highlights the % conservation at each nucleotide site. The other rows highlight the consensus sequence in fungi, plantae and metazoans respectively; conserved positions are shown as a dot.

### Soil samples

In autumn 2012, soil from 22 agricultural fields from different regions in Denmark was collected by taking 20 randomly distributed samples in each field from the upper 15 cm soil layer. These samples were pooled and mixed thoroughly. Subsamples of approximately 100 g were taken and freeze-dried for 48 hours. Dried samples were ground in a mixer mill (Retsch MM301, Haan, Germany) for 10 minutes, and 250 mg of soil was then used for DNA extraction using the PowerLyzer™ PowerSoil® DNA Isolation Kit (Mo Bio Laboratories, Carlsbad, CA, USA) according to the manufacturer’s instructions except that samples were further homogenized in a Geno/Grinder 2000 (SPEX CertiPrep, Metuchen, NJ, USA) at 1500 rpm for 3 × 30 seconds, instead of the commercial homogenizer recommended in the kit.

### PCR amplification and pyrosequencing

To generate amplicons for 454 pyrosequencing, primers NemF and 18Sr2b (Table 1) were used in a pre-amplification step followed by amplification with primers NF1 and 18Sr2b in a semi-nested procedure. NF1 and 18Sr2b were tag encoded using the forward primer 5′-CGTATCGCCTCCCTCGCGCCATCAG-MID-NF1-3′ and the reverse primer 5′-CTATGCGCCTTGCCAGCCCGCTCAG-18Sr2b-3′. Twenty-two 10-nucleotide MID primer tags for sample identification were randomly selected from the list of recommended MID primer tags from Eurofins MWG GmbH (Ebersberg, Germany). Primers were synthesized by Eurofins MWG GmbH. Reactions contained 1 × PCR reaction buffer, 1.5 mM MgCl_2_, 0.2 mM dNTPs, 1 μM of each primer, 1 U of GoTaq Flexi polymerase (Promega Corporation, Madison, USA) and 1 μl of DNA template diluted 1:10 (to app. 1 ng/μl) in a final volume of 25 μl. Amplifications were conducted in a GeneAmp PCR System 9700 thermal cycler (Applied Biosystems). Amplification with NemF and 18Sr2b was using an initial DNA denaturation step of 94°C for 5 min, followed by 20 cycles at 94°C for 30 sec, 53°C for 30 sec, 72°C for 1 min and a final elongation at 72°C for 10 min. The generated PCR product was diluted 1:10 and used as template in an amplification step with the tagged primers NF1 and 18Sr2b using the same conditions as in the first amplification except that annealing was at 58°C. The concentration of amplicons was estimated by analysis on a Nanodrop ND 1000 spectrophotometer (Thermo Scientific, Wilmington, DE, USA) according to the manufacturer’s instructions. Pooled amplicons were precipitated, redissolved and electrophoresed in 1.5% agarose gels, and a visible smear of PCR products at approximately 420 bp, corresponding to the expected size was cut from a gel and purified using a QIAquick Gel Extraction Kit (QIAGEN). The sample pool was sequenced by Eurofins MWG on a GS Junior 454 Sequencer (Roche Diagnostics).

### Data analysis

Sequences were processed in QIIME, version 1.7.0 [23] using the pipeline for analyzing 18S rDNA data. To de-noise flowgrams, reads mismatching with primer and MID sequences, PCR-based and sequencing errors and chimeras were removed using AmpliconNoise in combination with Perseus [24]. Default settings along with Uclust were used for *de novo* picking operational taxonomic units (OTUs) at 99% similarity as this level has previously been found to be the most appropriate for defining OTUs in Nematoda [11]. The Silva 108 release [12] was used as reference for taxonomic assignments of OTUs.

Representative sequences from each OTU were aligned together with the reference set of nematode sequences used in [9]. Alignments were done in MEGA5.22 using the Muscle algorithm with a gap penalty of 600 and a gap extension penalty of 60. The alignment was manually edited before a phylogenetic tree was constructed using the neighbor joining method.

### Availability of supporting data

The data set supporting the results of this article is available in the Dryad repository, doi:10.5061/dryad.0h653 [25].

## Additional files

Additional file 1: Table S1. Relative nematode quantities from this study compared to results from other studies. A comparison of nematode communities obtained in our study using NF1/18Sr2b and NemF/18Sr2b, respectively, is included. Additional file 2: Table S2. Relative distribution of sequences at order rank. Relative sequence distribution at order rank in the total dataset in each of the 22 soils. Additional file 3: Table S3. Identified OTUs with taxonomic identification. Reads distribution at OTU rank (99% identity) in the nematode dataset in each of the 22 soils. Additional file 4: Table S4. Representative sequences for each nematode OTU.
