# Supplementary material for: High-throughput sequencing of nematode communities from total soil DNA extractions
Source: BMC Ecol. 2015 Feb 12;15:3. doi: 10.1186/s12898-014-0034-4 (PMC4331302; doi:10.1186/s12898-014-0034-4)
Supplement: Additional file 1: Table S1. — Relative nematode quantities from this study compared to results from other studies. A comparison of nematode communities obtained in our study using NF1/18Sr2b and NemF/18Sr2b, respectively, is included. [file 12898_2014_34_MOESM1_ESM.docx]

Additional file 1: Table S1. Relative distribution (%) of nematode groups in agricultural soils investigated using morphology or sequencing.

| **Study** | **This study NemF** | **This study NF1** | **Darby et al. [10]** | **Li et al. [21]** | **Briar et al. [20]** | **Neher et al. [22]** | **Yeates et al. [23]** |
| --- | --- | --- | --- | --- | --- | --- | --- |
| Methodology | Sequencing | Sequencing | Sequencing | Morphology | Morphology | Morphology | Morphology |
| Location | Denmark | Denmark | Kansas | China | California | North Carolina | New Zealand |
| Cultivation practice | Annual crop | Annual crop | Grass plot | Annual crop | Annual crop | Annual crop | Perennial pasture |
| Araeolaimida | 6.5 | 4.0 | 0.05 | 0.2 | 0.2 |  | 10.0 |
| Ascaridida | 0.1 |  |  |  |  |  |  |
| Chromadorida | 0.9 | 3.4 | 1.6 | 0.1 |  | 1 |  |
| Desmodorida |  | 0.1 |  |  |  |  |  |
| Diplogasterida | 14.9 | 3.1 | 1.3 |  |  | 0.03 |  |
| Rhabditida | 17.3 | 7.1 | 84.7 | 10 | 18 | 44 | 37 |
| Tylenchida | 17.6 | 16.6 | 3.4 | 81 | 78 | 22 | 27 |
| Dorylaimida | 14.0 | 2.3 | 7.4 | 9 | 3 | 25 | 24 |
| Enoplida | 1.0 |  | 0.4 |  |  | 3 |  |
| Mermithida | 0.1 | 0.5 |  |  |  |  |  |
| Mononchida | 5.2 | 1.2 | 0.7 |  |  | 4 | 2 |
| Triplonchida | 1.3 | 1.7 | 0.2 |  |  | 0.01 |  |
| ^a^Others |  |  | 0.24 |  | 0.2 | 0.1 |  |
| ^b^Unclassified | 21.0 | 59.3 |  |  |  |  |  |

**Note:** a) others include group not listed in the table, b) unclassified includes nematodes that are unidentified at order level, however identified as nematodes, c) the proportion of nematode sequences in ‘This study NF1’ was 3 % of the total number.
